# Direct CNS delivery of proteins using thermosensitive liposome-in-gel carrier by heterotopic mucosal engrafting

**DOI:** 10.1371/journal.pone.0208122

**Published:** 2018-12-05

**Authors:** Grishma N. Pawar, Neha N. Parayath, Angela L. Nocera, Benjamin S. Bleier, Mansoor M. Amiji

**Affiliations:** 1 Department of Pharmaceutical Sciences, School of Pharmacy, Northeastern University, Boston, MA, United States of America; 2 Massachusetts Eye and Ear Infirmary, Harvard Medical School, Boston, MA, United States of America; Chapman University, UNITED STATES

## Abstract

Delivering therapeutics across the blood-brain barrier (BBB) for treating central nervous system (CNS) diseases is one of the biggest challenges today as the BBB limits the uptake of molecules greater than 500 Da into the CNS. Here we describe a novel trans-nasal mucosal drug delivery as an alternative to the intranasal drug delivery to overcome its limitations and deliver high molecular weight (HMW) therapeutics efficiently to the brain. This approach is based on human endoscopic skull base surgical techniques in which a surgical defect is repaired by engrafting semipermeable nasal mucosa over a skull base defect. Based on endoscopic skull based surgeries, our groups has developed a trans-nasal mucosal rodent model where we have evaluated the permeability of ovalbumin (45 kDa) as a model protein through the implanted mucosal graft for delivering HMW therapeutics to the brain. A thermo sensitive liposome-in-gel (LiG) system was developed for creating a drug depot allowing for a sustained release from the site of delivery to the brain through the implanted nasal graft. We would like to report this as an exploratory pilot study where we are using this novel surgical model to show that the implanted nasal mucosal graft and the LiG delivery system result in an efficient and a sustained brain delivery of HMW proteins. Hence, this study demonstrates that the trans-nasal mucosal engrafting technique could overcome the limitations for intranasal drug delivery and enable the uptake of HMW protein therapeutics into the CNS for the treatment of a wide range of neurodegenerative diseases.

## 1. Introduction

The number of people affected by neurodegenerative diseases such as Parkinson’s disease is expected to increase in the coming years [1] [2]. Additionally, it is estimated that the number of aged people suffering from neurodegeneration in the United States alone will double by 2030. Despite the significant societal and economic cost of these diseases, therapeutic strategies available to date remain largely symptomatic only and generally do not provide any disease-modifying benefits [3].

Even with the immense investment in this field, most biological neurotherapeutics have failed to reach the market as they cannot cross the BBB and the blood cerebrospinal-fluid barrier (BCSFB). Large protein based agents, including antibodies and growth factors, have demonstrated incredible potential in the treatment of Parkinson’s disease (PD), Alzheimer’s disease (AD), Amyotrophic lateral sclerosis (ALS) and many more [4]. A recent study demonstrated the clinical efficacy of adacanumab, a monoclonal antibody in patients with AD [5], (PRIME; ClinicalTrials.gov Identifier NCT0247780). However, systemically delivered proteins face an array of challenges in reaching the CNS as a result of rapid serum clearance, proteolytic degradation, and the presence of the BBB [6].

Currently there are no effective permanent strategies capable of permanently bypassing the BBB, a fact that has catalyzed numerous research efforts into overcoming this obstacle. Invasive strategies involve the physical disruption of the BBB, and the direct delivery of drug molecules into the CSF and brain parenchyma. This approach includes the use of stereotactically administered intraventricular, intrathecal, intracerebral injections [7]. Non-invasive strategies involve chemical or biological modification of drug molecules and utilization of the various receptors and transporters present on the endothelial cells of the BBB to deliver drugs to the brain parenchyma, thus preventing injury or trauma [8]. While each of these mentioned strategies is promising, they carry significant morbidity, are short-lived, and are difficult to scale.

Intranasal delivery to the brain is a non-invasive method that circumvents the BBB, which is an important barrier for CNS drug delivery. The intranasal pathway has been extensively explored as it takes advantage of the olfactory nerves that directly innervate the olfactory epithelium [9]. On intranasal administration, the administered drug molecules come in contact with the nasal mucosa, which is innervated by olfactory nerves, trigeminal nerves and is composed of olfactory epithelium and lamina propria [9]. The olfactory epithelium is composed of various cells like olfactory receptor neurons, basal cells, supporting cells, microvillar cells and is also composed of barriers like tight junctions, adherens junctions, gap junctions and desmosomes between the epithelial cells that determine the success of transport of administered drug molecules across the olfactory epithelium. [9–12]. The tight and the adherens junctions in the olfactory epithelia are composed of proteins like zona occludens and claudin-5 and they play an important role in providing barrier properties in the olfactory epithelium[11]. The transport of intranasally administered molecules takes place by either paracellular mechanisms (in between adjacent cells) or transcellular mechanisms (through endocytosis by passing through the cells) [9]. Hence the paracellular and transcellular transport mechanisms along with the presence of tight and adherens junctions in between adjacent cells in the olfactory epithelia affect the transport of drugs from nasal mucosa to the brain. Intranasal administration has several advantages like better patient compliance, reduced systemic exposure, avoiding the first pass metabolism leading to a better bioavailability [13]. Despite these advantages, there are several obstacles that prevent the clinical translation of this method. The drug contact with olfactory mucosa is highly variable between and within patients, which further results in variable drug absorption and poor distribution. Mucociliary clearance results in reduced mucosal residence time (15–20 minutes in contact with olfactory epithelium) that limits the drug absorption across the olfactory epithelium in the brain. Also the narrow “internal valve” in the anterior nose, limits drug distribution to the olfactory cleft, which again contributes to variable drug absorption in humans [14, 15]. Furthermore, the results from trans-nasal rodent models have failed to clinically translate due to significant deficit of olfactory mucosa in humans relative to rodents, the difference in the nasal cavity anatomy among different species, lack of animal diseases models that can replicate human diseases and identification of wrong pharmacological targets due to lack of understanding of physiology of the target in CNS diseases [16–19].

The limitations to trans-nasal drug delivery continue to persist despite of several developments and advantages in this field. One of the main reasons being that most of the published studies in humans do not describe the direct measurements of administered drugs in the CNS but the studies rather show indirect measurements as a proof of concept for direct nose-to-brain delivery. Several studies with intranasal administration of large molecules like corticotropin-releasing hormone, angiotensin II, melanocortin-melanocyte releasing hormone, diazepam, growth hormone releasing hormone, vasopressin have been published in recent years and have shown better therapeutic effects in comparison to subcutaneous and intravenous injections. But a large proportion of these published studies mainly measure the pharmacological effects or the elevated drug effects in cerebrospinal fluid (CSF) as a proof for direct nose-to-brain delivery. No studies have tried to measure the fractions of drug reaching the CNS, which is much less than 1% in animals [20]. Markus and van den Berg reviewed 104 human and animal studies prior to 2007. They used various criteria’s like dosing volumes, plasma concentrations, and pharmacokinetic measurements in plasma to compare intranasal administration to other routes of administration. Only 12 out of 104 studies passed their criteria to be effective in delivering drug intranasally. Hence it is difficult to clinically translate intranasal administration as means of efficiently delivering drugs to the CNS [21]. Hence, development of an alternative trans-nasal drug delivery into the brain could translate the current methods of CNS drug delivery.

In order to leverage the inherent advantages of trans-nasal delivery while overcoming its limitations, our group has developed a technique to create a semi-permeable mucosal conduit to the CNS in a rodent model using established endoscopic skull base surgical techniques. Endoscopic skull base surgery is a procedure, which is performed in clinics using an endoscope through nostrils. This surgical procedure is used to remove malignant and benign tumors at the junction between the nose and the brain while avoiding any external incisions. The lesions can be accessed by removing the intervening nasal mucosa, bone, dura and arachnoid membrane. This creates a direct window between the brain and the interior of the nasal cavity [22]. The window is repaired using nasal mucosal grafts, as the repairs are safe with no CSF leaks and show minimum long-term infections [23]. The nasal mucosal graft is highly porous and permeable and serves as a barrier between the body and the external environment. Hence, the nasal mucosal grafts have a strong immune defense mechanism, which reduces the chances of infections after implantation [24] [25]. Also as the surgery does not involve any external incisions, the patients can recover within less than 24 hours with less long-term side effects. As this surgical technique has proven to be safe, it is performed throughout the world using endoscopic instruments [26].

Based on the human endoscopic skull-based surgeries as explained above, our groups has designed an innovative platform in rodents as an alternative to the current trans nasal drug delivery for delivering high molecular weight therapeutics to the brain. The rodent model has been developed by creating a cranial window through the top of the rat head instead of the nose due to the smaller nasal surface available in rodents. This technique takes the advantage of creating a cranial window (craniotomy) similar to the ones performed in humans. The surgery involves removal of the bone, dura to expose the underlying brain similar to the endoscopic skull based surgeries performed in humans. The cranial window was then repaired using a nasal mucosal graft from a donor rat. No graft rejection was observed as the donor graft was from the same species of rats. A reservoir was then placed over the implanted graft to deliver the target molecule to the brain across the permeable nasal graft. Hence the main objective of developing a rodent model mimicking the endoscopic skull base surgeries in human was to create a drug conduit over a implanted permeable nasal graft to enhance the trans-nasal delivery through the nasal mucosa in the brain by overcoming the current trans-nasal delivery limitations.

Clinical adoption for successful delivery of high molecular weight therapeutic molecules requires the development of a carrier platform capable of prolonging mucosal residence time while simultaneously protecting the cargo from proteolytic degradation. Liposomes are highly lipophilic colloidal carriers that can encapsulate hydrophilic agents (proteins of interest) in the aqueous core and protect the payload from degradation [27],[28, 29]. The advantages of liposomes like high stability, better pharmacokinetic and biodistribution profile, drug release kinetics have made them one of the most successful delivery systems. These liposomes can improve transmucosal diffusion and may be engineered to provide a sustained release by incorporating them into a thermosensitive, mucoadherent Pluronic F-127 gel carrier. The liposomes-in-gel (LiG) formulation offers several advantages like tissue compatibility, ease of application, stability and sustained release of the payload over time [30],[31]. The purpose of this study was to test the feasibility of using a novel liposome-in-gel (LiG) carrier system to deliver proteins like ovalbumin, a surrogate high molecular weight protein to the brain using our dosing technique.

## 2. Materials and methods

### 2.1 Study design

**Fig 1:**
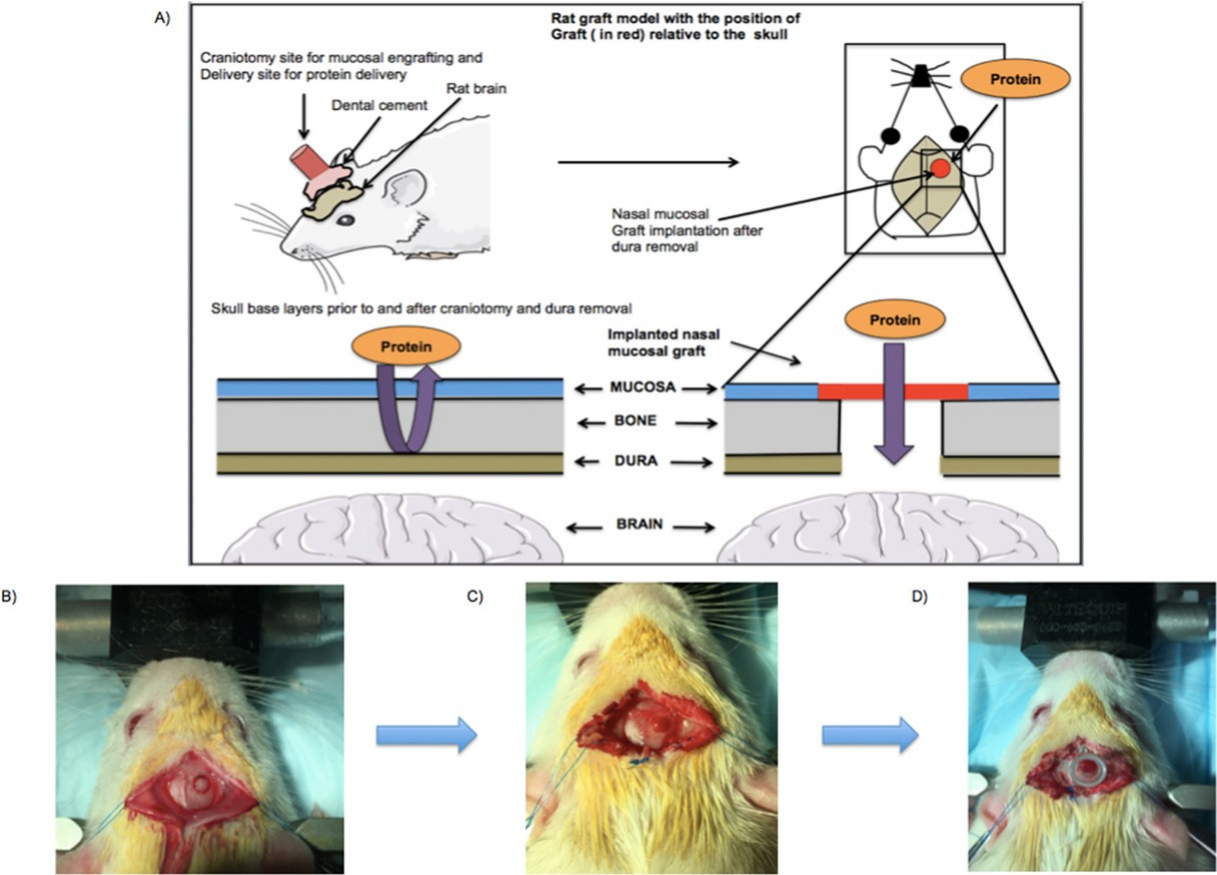
Rat model of heterotrophic mucosal engrafting technique. (A) A graphical representation of trans-nasal mucosal rodent model with implantation of nasal graft over the craniotomy for delivering high molecule weight proteins to the brain. (B) 3mm craniotomy was outlined using a surgical drill at 1.5 mm anterior posterior and -2mm medial-lateral to bregma. (C) The healed implanted mucosal graft 3 days after the engrafting procedure. (D) A propylene reservoir placed and secured over the graft.

We used an innovative heterotopic mucosal engrafting technique for delivering a representative high molecular weight protein (eg. ovalbumin, 45 kDa) to the rat brain in male Sprague-Dawley rats **(Fig 1A)**. After engraftment (described under surgical methods), the mucosa was initially exposed to Cy5-labeled ovalbumin and ovalbumin in saline. The uptake of the Cy5-labeled ovalbumin in solution was quantitatively determined after 72 hours using an ICYTE imaging cytometer (CompuCyte Corp., Cambridge, MA). The uptake of ovalbumin in solution in the rat brain was then determined after 48 and 72 hours quantitatively using commercially available ovalbumin specific (Biomatik) Elisa kit. A thermosensitive LiG formulation was then designed and formulated to provide sustained delivery to the rat brain over 72 hours. The uptake of Cy5-labeled ovalbumin LiG and ovalbumin LiG was determined after 72 hours using similar techniques as mentioned above.

#### 2.1.1. Statistical design and analysis

The primary statistical analyses were done using GraphPad Prism (version 7.00, La Jolla California, USA, www.graphpad.com).

*Rodent studies*: Two statistical tests were employed for rodent studies. The first test employed was a 3 x 4 between (3 Treatment Groups) and within (4 Brain Parts) subject Anova. Significant main or interaction effects were followed up with Tukey post hoc tests comparing cell means to pinpoint the specific nature of the overall effects. The second test employed was between subject Anovas followed by Tukey post hoc tests of differences between treatment groups.

A sample size of n = 2 was used for each treatment group (untreated), Ovalbumin in saline (48 and 72 hours) and Ovalbumin LiG (48 and 72 hours).

*Human studies*: Two human subjects were tested in this study. The statistical analysis employed was a multifactor 2 x 6 repeated measures Anova design. The interaction between two formulations and time points (0, 1, 1.5, 2, 2.5 and 3 hours) at which aliquots were measured was of primary interest. Post hoc paired T tests were applied to determine differences between the two vehicles (cy5-labeled ovalbumin in saline and LiG) at each time point.

#### 2.1.2. Ethics statements

All the animal procedures were approved by the Northeastern University Institutional Animal Care and Use Committee (Protocol number: 180101-R) that was approved in January, 2018. For mucosal permeability studies, de-identified human septal mucosal tissue samples were obtained from Mass Eye and Ear Infirmary (MEEI). An Investigational Review Board (IRB) at MEEI has approved the protocol for harvesting the tissue samples in May, 2018. Northeastern University IRB has also approved protocol number 16-06-03 to assess mucosal permeability with the de-identified tissue obtained from MEEI in May, 2018.

### 2.2 Surgical methods

#### 2.2.1 Donor graft

The Sprague-Dawley rats used in the study were ordered from Charles River, Kingston, MA, USA. All surgeries were performed under isoflurane anesthesia and all efforts were made to minimize suffering. All the rats were housed in a climate controlled room on a 12/12-hour light/dark cycle and were provided with food and water ad libitum. All the surgeries were performed during the rat’s light cycle. For the heterotopic mucosal engrafting procedure, a donor rat was used to harvest the nasal septum according to the methods previously described in [25, 32, 33]. Briefly, a donor rat was euthanized by carbon dioxide and surgical scissors were used to remove the skin from the nasal dorsum. A surgical drill and scissors were used to remove and isolate a unilateral septal mucoperichondrial graft, which was stored in saline solution for a maximum of two hours.

#### 2.2.2 Graft implantation

The experimental rat (250–300 grams) was anesthetized using isoflurane placed in a stereotaxic frame under an operating microscope. The surgical site was sterilized using povidone iodine and alcohol swabs. A sagittal incision was made from the level of midorbit to the occiput using a scalpel. Bilateral skin flaps were then elevated exposing the pericranium. A scalpel was used to clear the pericranium from the intended craniotomy site (1.5 mm anterior-posterior and 2 mm medial-lateral to bregma). A surgical drill was used to create a 3 mm craniotomy leaving the underlying dura intact **(Fig 1B).** The underlying dura and arachnoid were then removed leaving the underlying pia matter undisrupted. The harvested graft from the donor rat was then implanted over the craniotomy such that the basolateral membrane of the graft faced the exposed pia matter. A piece of sterile nitrile was placed over the graft to prevent adhesion to surrounding tissue. The skin flaps were the sutured back and the rat was left to engraft for 3 days. After 3 days the skin flaps were again reflected carefully without disrupting the underlying implanted mucosal graft. The graft was then inspected using a dissection microscope to ensure viability and circumferential engraftment **(Fig 1C).**

#### *2*.*2*.3 Reservoir placement

A 250 μl polypropylene reservoir was placed over the mucosal graft such that the reservoir had good contact with the surrounding skull. The reservoir was attached to the skull using cyanoacrylate and tested for leaks using sterile saline. After implantation of a screw (Morris Precision screws and parts– 000x 3/32 Flat self tap screws), dental cement from Stoelting was applied to the skull to fix the reservoir **(Fig 1D).**

### 2.3 Qualitative and quantitative evaluations of transmucosal delivery

#### 2.3.1 Cy5-labeling of ovalbumin

Chicken egg ovalbumin (Sigma Aldrich) and sulfo Cy5-NHS ester dye (Lumiprobe) was used for labeling ovalbumin with Cy5. The ratio of ovalbumin and Cy5 required for labeling was calculated according to the manufacturer's protocol. Briefly, ovalbumin and Cy5 were dissolved in saline and stirred overnight in the dark. After 24 hours, the ovalbumin Cy5 conjugate solution was dialyzed (12000–14000 Da dialysis membrane) for 18 hours in 1X PBS followed by overnight dialysis in deionized water to remove unconjugated protein and dye. The conjugate solution was freeze-dried overnight and utilized for dosing animals.

#### 2.3.2 Dosing of cy5-labeled ovalbumin and unlabeled ovalbumin in rat brain

For Cy5-labeled ovalbumin dosing 6 μg dissolved in 60 μl saline (n = 1) was placed in the reservoir. The rats were sacrificed after 72 hours. For ovalbumin dosing alone, 50 μg of ovalbumin in 50 μl of saline (n = 2) was placed in the reservoir. The rats dosed were sacrificed after 48 and 72 hours.

Among the Cy5-labeled ovalbumin group, brains were flash frozen and placed in OCT solution at -80°C. The brain was then sliced into 50-micron sections using a cryotome and observed using an ICYTE imaging cytometer (CompuCyte Corp., Cambridge, MA) for quantitative uptake of Cy5-labeled ovalbumin. Among the ovalbumin group, brain was divided into 4 equal parts using a rat brain coronal precision brain slicer (Braintree Scientific, Braintree, MA) starting from anterior to the posterior part of the brain. Each part was weighed and homogenized in tissue lysis buffer (50 mM Tris-HCl, pH 7.5, 50 nM NaCl and 0.5% v/v IGEPAL CA-640) containing the EDTA-free Protease inhibitor cocktail tablet (Sigma-Aldrich, St. Louis, MO). Homogenate from each section was centrifuged at 14,000 g for 30 minutes and the supernatant was collected, and the ovalbumin uptake was quantified by enzyme linked immunosorbent assay (ELISA, Biomatik, Wilmington, DE).

### 2.4 Evaluation of transmucosal delivery using cationic and anionic LiG formulations

#### 2.4.1 Preparation of cationic and anionic liposomes

The cationic lipid film was prepared using DOTAP (1,2-dioleoyl-3-trimethylammonium-propane chloride salt) a cationic lipid, cholesterol (stabilizer), and DPPC (1,2-dipalmitoyl-sn-glycero-3-phosphocholine) a neutral lipid in a 5:3:5 molar ratio. For the lipid film, a stock concentration of each lipid was made in chloroform and 1ml of each lipid was added to a Sigma-Aldrich ST/NS14/20 10 ml round-bottom flask attached to a Rotavap (IKA works Inc. Wilmington, NC-28405, Model RV 10 C S99), and allowed to rotate at 100 rpm in a water bath at room temperature (RT). An anionic lipid film was formed using 2.37 mg DPPC (1,2-dipalmitoyl-sn-glycero-3-phosphocholine) a neutral lipid, 2.35 mg cholesterol (stabilizer) and 0.9 mg 1,2-distearoyl-sn-glycero-3-phosphoethanolamine-N [amino (polyethylene glycol)-2000] ammonium salt (DSPE-PEG 2000) dissolved in 5 ml chloroform in a Sigma-Aldrich ST/NS14/20 10 ml round-bottom flask attached to a Rotavap, and allowed to rotate at 100 rpm in a water bath at room temperature. Following chloroform evaporation, the thin lipid film at the bottom of the flask was dried overnight in vacuum to remove the solvent, and the film (cationic and anionic) was subsequently hydrated with 250 ug/ml of Cy5-labeled ovalbumin and ovalbumin solution in saline as starting concentration, followed by additional vortexing for 1 minute. After hydration of the lipid film, the liposomal preparation was placed on ice for two minutes, vortexed, placed in a water bath at 37 degrees for two minutes, and vortexed again. Five such freeze-thaw cycles were performed.

The liposomal preparation was then probe-sonicated for five minutes on ice. The mixture was ultracentrifuged in a Beckman-Coulter ultracentrifuge at 100,000 rpm for 1 hour to separate the protein-encapsulated liposomes from unencapsulated protein. The pelleted liposomes were suspended in saline to get a liposomal solution, extruded through 800nm, 400nm, and 200 nm membranes using an extruder (Avanti Lipids, Alabaster, AL) and the supernatant was used for further analysis. Encapsulation efficiency was determined with the use of an indirect method. In the indirect method, the amount of protein in the supernatant obtained after ultracentrifugation was measured using the Pierce BCA assay kit (Thermo Fisher Scientific) for ovalbumin and was subtracted from the starting amount of the protein to get the total protein encapsulated in liposomes. Extruded liposomes were characterized for hydrodynamic diameter, polydispersity index (PDI), and surface charge (zeta potential) using a Zetasizer (Nano-ZS90, Malvern Instruments, Inc Westborough MA). Liposomal morphology was determined by the transmission electron microscopy (TEM).

#### 2.4.2 Preparation of cy5-labeled ovalbumin and ovalbumin LiG

Pluronic F-127 (BASF Corp., Florham Park, NJ) was used to prepare a thermosensitive LiG system. The Pluronic F-127 gel remains liquid at 4°C but turns into a viscous gel at RT. Hence the entire procedure was performed at 4°C. A 30% (w/v) Pluronic F-127 aqueous solution was prepared by slowly adding 3 grams of Pluronic F-127 in 10 ml of saline while stirring continuously at 4°C until completely dissolved. The Pluronic F-127 solution was left at 4°C to remove air bubbles and the Cy5-labeled ovalbumin encapsulated in anionic or cationic liposomes as well as unlabeled ovalbumin encapsulated in cationic liposomes were added to the solution at 4°C and stirred for 20 minutes to form a homogenous LiG system. The final concentration of either Cy5-labeled ovalbumin or unlabeled ovalbumin in the LiG was based on the dosing required for the animal experiments.

#### 2.4.3 Cy5-labeled ovalbumin and ovalbumin LiG dosing in rat brain

For Cy5-labeled ovalbumin anionic and cationic LiG dosing, the liposomes were suspended in 30% (w/v) Pluronic F-127 such that 60 μl cationic and anionic LiG dose contained 6 μg of Cy5-labeled ovalbumin. Cy5-labeled ovalbumin in saline (6 μg in 60 μl saline) and Cy5-labeled ovalbumin in Pluronic F-127 gel (6 μg in 60 μl of 30% Pluronic F-127 gel) were used as controls. For ovalbumin cationic LiG dosing, cationic liposomes were suspended in 30% Pluronic F-127 gel such that 170 μl cationic LiG dose contained 50 μg of ovalbumin (n = 2). The rat brains were further processed for qualitative and quantitative uptake as mentioned in Section 2.3.2 above.

### 2.5 Permeability of ovalbumin in LiG across human nasal mucosal tissue

De-identified tissue samples were harvested from patients undergoing endoscopic sinonasal surgery. The mucosa was mounted in a self-contained Ussing’s chamber (Warner Instruments, LLC Hamden, CT 06514, Model U2500) and incubated in transport medium (375 ml HBSS (Hank’s balanced salt solution), 892.5 mg HEPES 10 mM (4-(2-hydroxyethyl)-1-piperazineethanesulfonic acid) and 675 mg D (+) glucose 10 mM) for 10 minutes at 37°C. After equilibrating in the transport medium, the chamber facing the apical side of the mucosa was emptied and was filled with 250 μg of Cy5-labeled ovalbumin in 3 ml 1X PBS or 250 μg of Cy5-labeled ovalbumin liposomes suspended in 1 ml 1X PBS and 2 ml of 30% Pluronic F-127 gel and the basolateral side was filled with 3 ml of transport medium. The nasal mucosa was then incubated for 3 hours with the formulations with a continuous supply of carbogen. After 1, 1.5, 2, 2.5 and 3 hours a 200 μl sample was aliquoted from the basolateral side. Fluorescence was measured using a Biotek plate reader. The apparent permeability coefficient was calculated using the equation

$$\text{Papp}=\left(\text{dQ/dt}\right)/\left({\text{C}}_{\text{0}}\text{X}\;\text{A}\right)$$

where dQ/dt is the transport rate and is defined by the slope obtained from linear regression of the amount (nanograms) transported on the basolateral side. C_0_ is the initial concentration of Cy5-labeled ovalbumin in saline and LiG on the apical side and A is surface area of the tissue.

## 3. Results and discussion

### 3.1 *In vivo* distribution of cy5-labeled ovalbumin and unlabeled ovalbumin in saline

**Fig 2:**
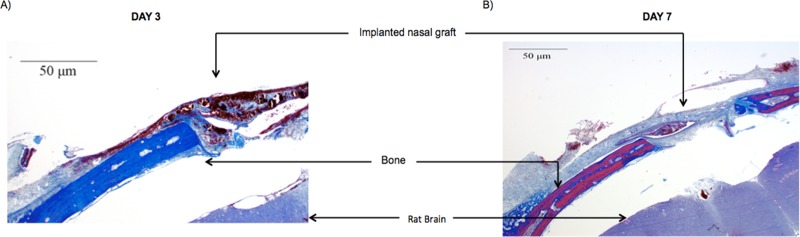
**Mason’s Trichome staining of the intact mucosal graft over the craniotomy with no sign of infections at** (A) Day 3 and (B) Day 7.

To determine the impact of trans-nasal mucosal delivery on the uptake of high molecular weight proteins, we developed a rat model to mimic the human skull base surgery as described in materials and methods section **2.2.** The mucosal engrafting surgery was well tolerated in rat and there was no evidence of infections. Mason’s Trichome staining showed that the implanted grafts were intact, and the craniotomy was completely covered with the graft after day 3 and day 7 **(Fig 2).**

**Fig 3:**
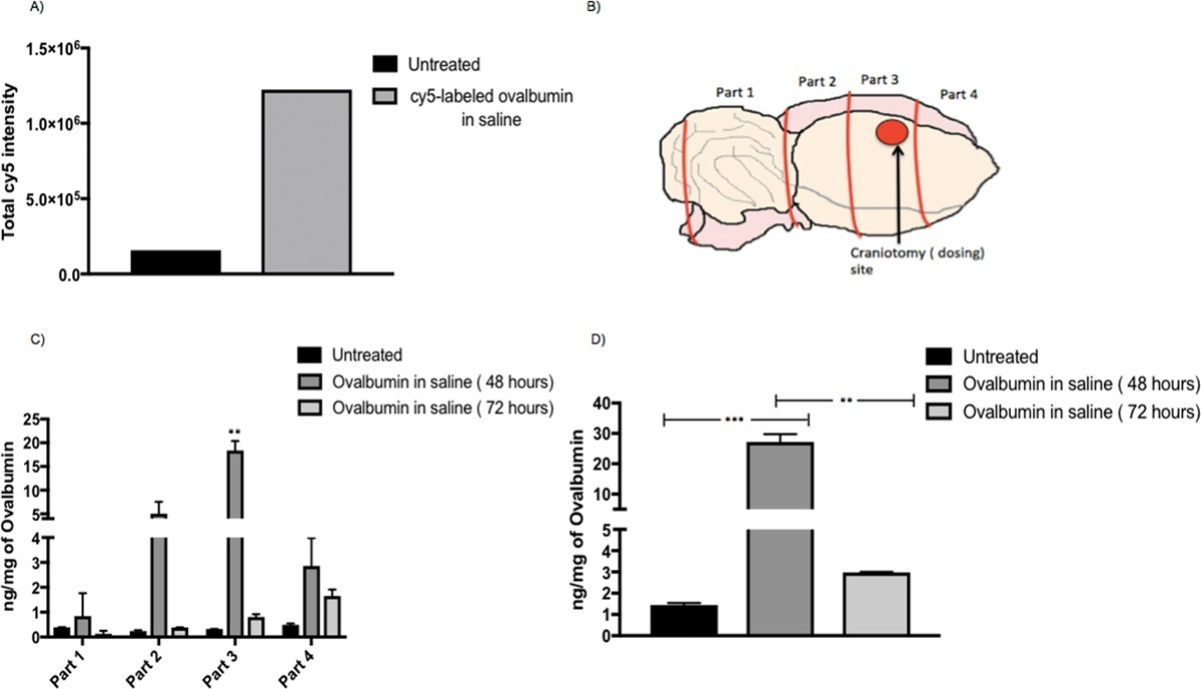
Qualitative and Quantitative uptake of cy5-labeled ovalbumin and ovalbumin in saline in rat brain using mucosal engrafting technique. (A) Imaging cytometry quantified data from the four selected regions for untreated and Cy5-labeled ovalbumin in saline treated rats (n = 1) using equation total Cy5 intensity = area of Cy5 in selected region in each section * total Cy5 intensity in each selected region in each section. (B) Rat brain cut into 4 equal parts for ELISA with part 3 being the craniotomy site. (C) Ovalbumin detected by ELISA for treatment groups: Untreated (n = 2), ovalbumin saline 48 hours (n = 2), ovalbumin saline 72 hours (n = 2) in the 4 isolated parts of the brain. A 2-way 3 x 4 between and within subjects Anova indicated a significant overall interaction (**** p<0.0001, F (6,9) = 35.75) between different parts (4 brain parts) of the brain and the different treatment groups (untreated, ovalbumin in saline 48 and 72 hours). Post hoc tukey’s tests were further applied to determine differences in ovalbumin uptake in different brain parts. Significant group effects were only found in part 3 where Ovalbumin in saline 48 hours showed significantly greater uptake as compared to untreated rats (** p = 0.0011, DF = 3) and ovalbumin in saline 72 hours(** p = 0.0012, DF = 3). (D) Total ovalbumin (four parts combined) detected by ELISA for treatment groups: Untreated ovalbumin saline 48 hours, ovalbumin saline 72 hours. Ordinary between subjects one way Anova with Tukey’s tests was applied. Ovalbumin in saline (48 hours) showed significantly greater uptake as compared to untreated (*** p = 0.0009, DF = 3) and ovalbumin in saline (72 hours) (** p = 0.0011, DF = 3). A sample size of n = 2 was used for each treatment group.

Based on the imaging cytometry data, the sections from rat brain treated with Cy5-labeled ovalbumin formulation showed high Cy5 fluorescence intensity (**S1 Fig**). Thus, the mucosal engrafting techniques led to a significant uptake of Cy5-labeled ovalbumin in rat brain **(Fig 3A).**

The total uptake of ovalbumin in rat brain was determined by dividing the rat brain into four equal parts using a precision brain slicer (Braintree scientific) (**Fig 3B**). This was done to determine the uptake of ovalbumin in four different parts of the brain based on their distance from the site of delivery (craniotomy) with part 2 being exactly below the site of delivery and part 4 being farthest from the site of delivery. The results in **Fig 3C** shows that there was a significant uptake of ovalbumin in part 3 for ovalbumin in saline 48 hours as compared to untreated rats confirming maximum uptake near the site of the craniotomy. The results in **Fig 3D** showed that the total ovalbumin uptake (four parts combined) was significantly more at 48 hours as compared to ovalbumin in saline 72 hours and untreated rats. It can be concluded that at longer time points, such as, by 72 hours the uptake of ovalbumin in the rat brain decreases when compared with 48 hours. This could be attributed to the faster diffusion pattern of the saline formulation when instilled in the reservoir directly above the craniotomy.

### 3.2 *In vivo* distribution of cy5-Labeled ovalbumin encapsulated in anionic and cationic LiG formulations and unlabeled ovalbumin encapsulated in cationic LiG formulations

**Fig 4:**
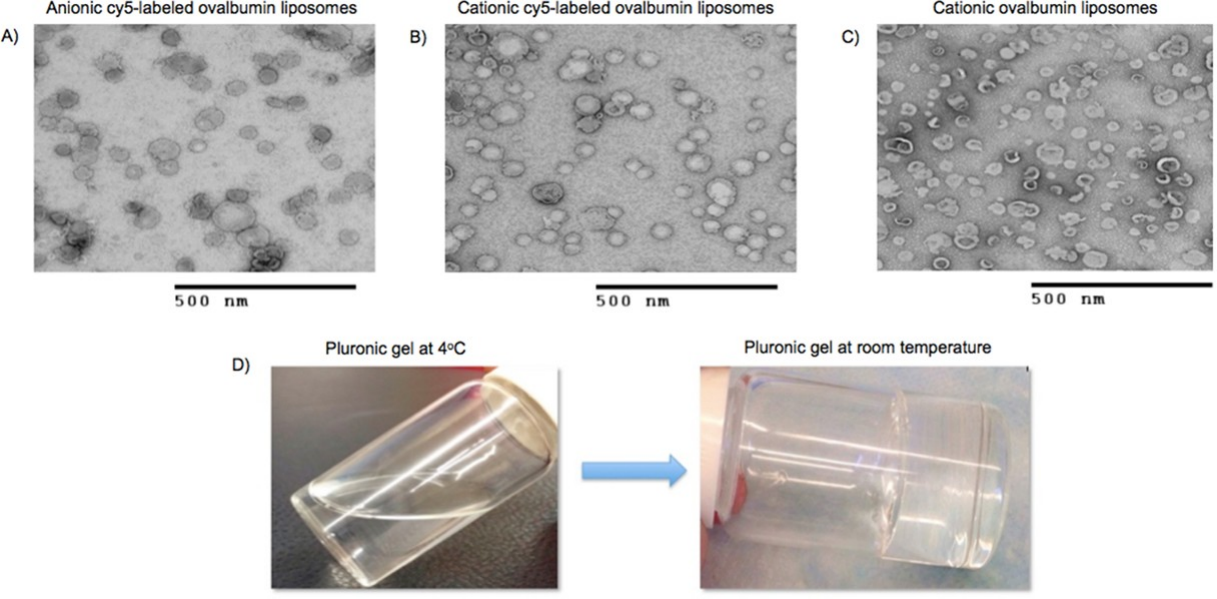
Transmission electron microscopy images of formulated liposomes. (A) Anionic Cy5-labeled ovalbumin liposomes (B) cationic Cy5-labeled ovalbumin liposomes (C) and cationic unlabeled ovalbumin liposomes (D) A 30% (w/v) thermo-sensitive Pluronic F-127 solution at 4°C and gel at room temperature.

The formulated Cy5-labeled ovalbumin cationic and anionic liposomes and unlabeled ovalbumin cationic liposomes were characterized for their size, polydispersity index, surface charge, and TEM was used for determining the structure of liposomes **(Table 1 and Fig 4A, 4B and 4C).** A negative stain (uranyl acetate) was used to stain the liposomes.

**Table 1:** Characterization data (average size, PDI, surface charge and percent encapsulation efficiency) for anionic cy5-labeled ovalbumin liposomes, cationic Cy5-labeled ovalbumin and cationic unlabeled ovalbumin liposomes (n = 3).

Liposome sample	Average size (nm)	Polydispersity index (PDI)	Average charge (mV)	Percent encapsulation efficiency
Anionic cy5-labeled ovalbumin liposomes	148.9 ± 1.3	0.2 ± 0.08	-1.7 ± 0.01	73.7 ± 13.7
Cationic cy5-labeled ovalbumin liposomes	181.8 ± 12.2	0.3 ± 0.05	+ 30.3 ± 1.2	97 ± 0.2
Cationic unlabeled ovalbumin liposomes	221.6 ± 22.8	0.2 ± 0.3	+22.7 ± 1.5	85 ± 0.2

**Fig 5:**
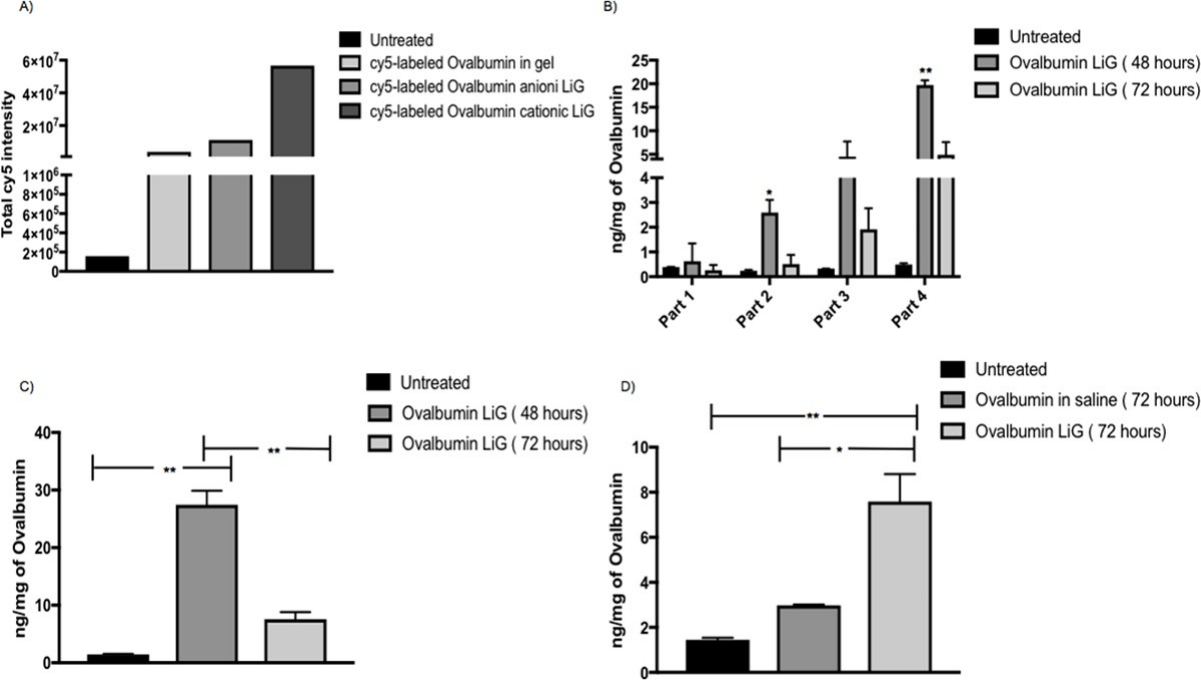
Quantitative uptake of ovalbumin cationic LiG in rat brain using mucosal engrafting technique. (A) ICYTE quantified data from the four selected regions for untreated, Cy5- labeled ovalbumin in Pluronic F-127 gel, Cy5-labeled ovalbumin anionic LiG and Cy5-labeled ovalbumin cationic LiG (n = 1) using equation total Cy5 intensity = area of Cy5 in selected region in each section * total Cy5 intensity in each selected region in each section. (B) Ovalbumin detected by ELISA for treatment groups: Untreated (n = 2), ovalbumin LiG 48 hours (n = 2), ovalbumin LiG 72 hours (n = 2) in the 4 isolated parts of the brain. A 3 x 4 between and within subjects 2 way Anova was applied and a significant overall interaction (**** p<0.0001, F (6,9) = 20.15) was found between different parts (4 brain parts) of the brain and the different treatment groups (untreated, ovalbumin in LiG 48 and 72 hours). A post hoc tukey’s test was further applied to determine difference in ovalbumin uptake in different brain parts. Ovalbumin LiG 48 hours shows significant uptake in part 2 as compared to untreated (* p = 0.0154, DF = 3) and ovalbumin LiG 72 hours (* p = 0.0217, DF = 3). Ovalbumin LiG 48 hours also shows significant uptake in part 4 as compared to untreated (** p = 0.0028, DF = 3) and ovalbumin LiG 72 hours (**p = 0.0059, DF = 3). (C) Total Ovalbumin detected (four parts combined) by ELISA for treatment groups: Untreated (n = 2), ovalbumin LiG 48 hours (n = 2), ovalbumin LiG 72 hours (n = 2). Ovalbumin LiG (48 hours) shows significant uptake as compared to untreated (** p = 0.0010, DF = 3) and ovalbumin LiG (72 hours) (** p = 0.0022, DF = 3). (D) Ovalbumin LiG (72 hours) shows significant uptake as compared to untreated (** p = 0.0065, DF = 3) and ovalbumin in saline (72 hours) (* p = 0.0147, DF = 3). A sample size of n = 2 was used for each treatment group.

After hardening of the dental cement, the rats were dosed with LiG formulations as mentioned in **section 2.4.3**. All the animals were sacrificed after 72 hours and the brains were processed as mentioned in **section 2.3.2**. From **Fig 5A** it was observed that Cy5-labeled cationic liposome-in-gel had the maximum uptake as compared to the anionic LiG and the other control groups. Hence, it was decided to perform further experiments with cationic LiG only.

Rats were dosed with cationic ovalbumin LiG as mentioned in **section 2.4.3.** The animals in each group were sacrificed after 48 and 72 hours and the uptake were determined using protein specific ELISA. From **Fig 5B** it can be seen that ovalbumin LiG showed significantly better uptake in parts 2 and 4 as compared to untreated rats again confirming maximum uptake near the site of craniotomy. From **Fig 5C** it can be seen that there was a significant uptake of ovalbumin cationic LiG in rat brain at 48 as compared to 72 hours which can again be attributed to the fast diffusion pattern of the formulation from the reservoir. However, from **Fig 5D** ovalbumin LiG resulted in a significant uptake in rat brain at 72 hours as compared to untreated and ovalbumin in saline at 72 hours. Hence it can be concluded that the cationic LiG formulation helps in a time-dependent controlled release as the amount of ovalbumin detected in rat brain at 72 hours for LiG formulation was significantly more than the saline formulation and untreated rats.

### 3.3 Permeability of cy5-labeled ovalbumin LiG through human nasal mucosal tissue

**Fig 6:**
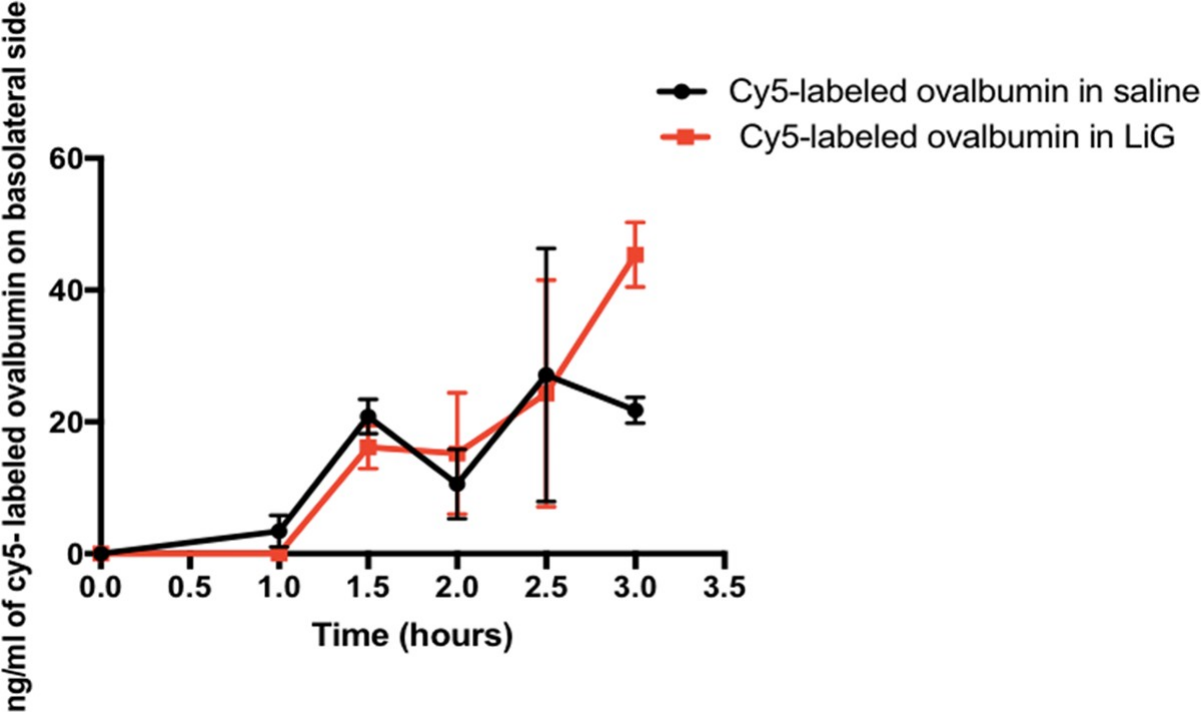
Diffusion of Cy5-labeled ovalbumin in saline and LiG through human nasal mucosal tissue for up to 3 hours. A 2x6 multifactor repeated measures anova design was applied and a significant overall interaction between the timepoints and vehicles (cy5-labeled ovalbumin in saline and LiG) was obtained (* p<0.05, F (5,5) = 8.349). Post hoc paired T tests were applied to determine the difference between vehicles at each time point. No significant difference was found.A sample size of n = 2 was used for each treatment group.

The uptake of Cy5-labeled ovalbumin through human nasal mucosa was studied using Ussing’s chamber. Sample was collected from the basolateral side of the chamber to measure the fluorescence intensity at defined time-points. Both Cy5-labeled ovalbumin in saline and cationic LiG can permeate through the graft from apical to the basolateral side and there was an increase in the diffusion from time zero to 3 hours though not significant. Also at 3 hours ovalbumin LiG showed increase in uptake as compared to cy5-labeled ovalbumin in saline. However no statistical significance was found. It can be observed that the overall significant interaction is sensitive to different patterns of means across time but no individual post hoc tests are significant for any given time point due to lower number of samples used in this study. The Cy5-labeled ovalbumin in saline had an apparent permeability coefficient of 2.6 x 10^−5^ cm/hour and Cy5-labeled ovalbumin LiG had an apparent permeability coefficient of 3.5 x 10^−5^ cm/hour. Hence this shows that the human nasal graft is permeable to both formulations for up to 3 hours **(Fig 6).** The study was conducted up to 3 hours due to ex vivo tissue viability issues.

## 4. Discussion

The purpose of this study was to determine whether the LiG formulation could be used to encapsulate surrogate protein therapeutics and enhance delivery to the brain using our previously described transmucosal grafting technique. We used ovalbumin (45 kDa) as a model protein, as its successful delivery provides a proof-of-concept for other protein-based therapeutics, which have already been explored in neurodegenerative diseases.

The described rodent extra-cranial graft model we utilized is based on widely used endoscopic skull base reconstructive techniques. While the successful transmucosal delivery of high molecular weight protein based neurotrophic factors has previously been established [25], [34, 35], biological therapeutics are highly susceptible to degradation by proteases, DNase’s and RNase’s. Furthermore, solutions delivered intranasally are subject to variable exposure to the mucosal graft leading to unpredictable pharmacokinetics. Consequently, the transmucosal paradigm requires a delivery platform capable of protecting the biologic cargo from such degradation while simultaneously enhancing mucosal contact and retention.

Our results from **Fig 3**demonstrate that high molecular weight proteins (eg. ovalbumin, 45 kDa) can be delivered to the brain in significant amounts as compared to untreated rats, further validating the transmucosal delivery route. Our results from **Fig 4**demonstrate that both cationic and anionic liposomes can be formulated using both Cy5-labeled ovalbumin and ovalbumin alone with cationic liposomes having maximum encapsulation efficiency. The higher encapsulation efficiency for cationic liposomes can be attributed to the electrostatic complexation between the positively charged liposomes and the negatively charged ovalbumin at pH 7 (isoelectric point for ovalbumin is 4.54) [36]. Our results from **Fig 5A** indicate that the cationic LiG has better uptake than the anionic LiG. Results from **Fig 5D** demonstrate that the encapsulation of the ovalbumin in LiG enhances transmucosal delivery over 72 hours thereby validating this delivery platform and setting the stage for delivery of other high molecular weight protein based neurodegenerative therapies. Results from **Fig 6**demonstrate that the diffusion through human nasal mucosa approximates that of our rat model. Furthermore, our results show that the use of the LiG carrier does not impair ovalbumin diffusion over short time frames of less than 3 hours. This experiment was not performed for longer time points due to human explant viability issues.

## 5. Conclusions

We would like to report this study as an exploratory pilot study through which our findings confirm that the described mucosal engrafting technique can be used to deliver representative high molecular weight proteins to the brain by overcoming the limitations of intranasal drug delivery. Our results therefore validate the rat as a novel model of direct transmucosal delivery and open the door to testing biologically active and clinically relevant protein based agents such as antibodies and neurotrophic factors for the treatment of neurodegenerative disease. Additionally, the use of the LiG delivery platform improved the delivery of our surrogate protein, ovalbumin, emphasizing the effectiveness of LiG formulations to protect the drug from degradation and act as a depot for drug for sustained release.

## Supporting information

S1 Fig Qualitative uptake of cy5-labeled ovalbumin in saline, pluronic gel, anionic LiG and cationic LiG in rat brain as compared to untreated rats using imaging cytometry. (TIF)

S1 File Raw experimental dataset. (PDF)
