# Supplementary figures and images for: Direct CNS delivery of proteins using thermosensitive liposome-in-gel carrier by heterotopic mucosal engrafting
Source: PLoS One. 2018 Dec 5;13(12):e0208122. doi: 10.1371/journal.pone.0208122 (PMC6281301; doi:10.1371/journal.pone.0208122)

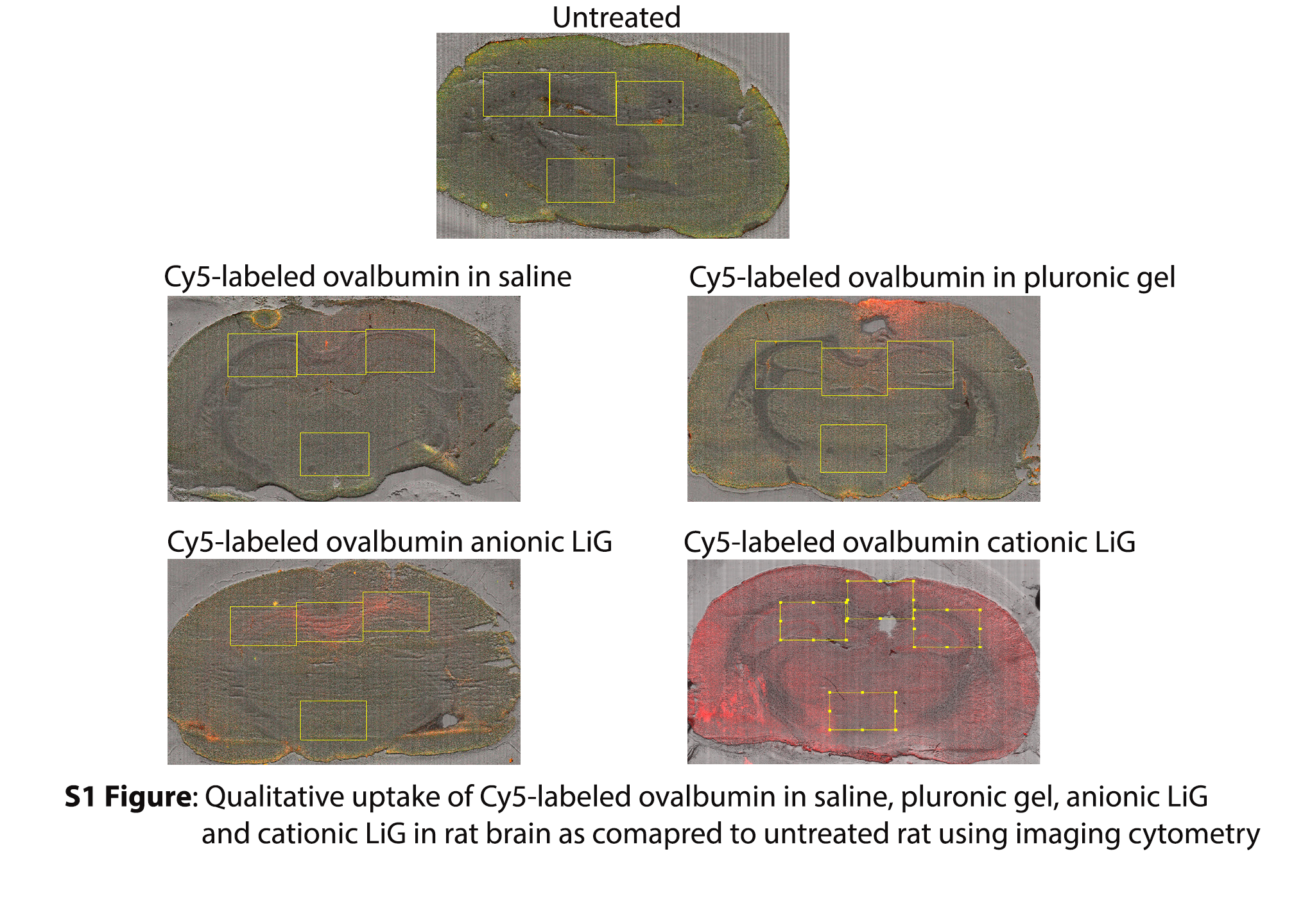

Supplement: S1 Fig — (TIF) [file pone.0208122.s001.tif]
