# Supplementary material for: Direct CNS delivery of proteins using thermosensitive liposome-in-gel carrier by heterotopic mucosal engrafting
Source: PLoS One. 2018 Dec 5;13(12):e0208122. doi: 10.1371/journal.pone.0208122 (PMC6281301; doi:10.1371/journal.pone.0208122)
Supplement: S1 File — (PDF) [file pone.0208122.s002.pdf]

Explanatory Notes and Guidance
Recommended Repositories
Repository Inclusion Criteria
FAQs for Data Policy
PLOS Data Advisory Board
Give Feedback

Explanatory Notes and Guidance

A compilation of frequently asked questions about the PLOS Data Policy is available and is updated periodically.

Definition of data that must be shared

PLOS defines the "minimal data set" to consist of the data set used to reach the conclusions drawn in the manuscript with related metadata and methods, and any additional data required to replicate the reported study findings in their entirety. Authors do not need to submit their entire data set if only a portion of the data were used in the reported study. Also, authors do not need to submit the raw data collected during an investigation if the standard in the field is to share data that have been processed.

Please note that PLOS does not permit references to "data not shown." Authors should provide the relevant data within the manuscript, the Supporting Information files, or in a public repository. If the data are not a core part of the research study being presented, we ask that authors remove any references to these data.

Guidance on sharing data sets that derive from clinical studies or other work involving human participants

For studies involving human participants, data must be handled so as to not compromise study participants' privacy. PLOS recommends that researchers follow established guidance and applicable local laws in ensuring they do not compromise participant privacy. Resources which researchers may consult for guidance include:

- US National Institutes of Health: Protecting the Rights and Privacy of Human Subjects
- Canadian Institutes of Health Research Best Practices for Protecting Privacy in Health Research
- UK Data Archive: Anonymisation Overview
- Australian National Data Service: Ethics, Consent and Data Sharing

Steps necessary to protect privacy may include de-identification, blocking portions of the database, or license agreements directed specifically at privacy concerns. Authors should indicate, as part of the ethics statement, the ways in which the study participants' privacy was preserved. If license agreements apply, authors should note the process necessary for other researchers to obtain a license.
